# Handedness Dependent Electromagnetically Induced Transparency in Hybrid Chiral Metamaterials

**DOI:** 10.1038/srep12224

**Published:** 2015-07-17

**Authors:** Lei Kang, Zhi Hao Jiang, Taiwei Yue, Douglas H. Werner

**Affiliations:** 1 Department of Electrical Engineering, The Pennsylvania State University, University Park, PA, 16802 USA

## Abstract

We provide the first experimental demonstration of the handedness dependent electromagnetically induced transparency (EIT) in chiral metamaterials during the interaction with circularly polarized waves. The observed chiral-sensitive EIT phenomena arise from the coherent excitation of a non-radiative mode in the component split ring resonators (SRRs) produced by the corresponding Born−Kuhn type (radiative) resonators that are responsible for the pronounced chirality. The coherent coupling, which is dominated by the bonding and antibonding resonances of the Born−Kuhn type resonators, leads to an extremely steep dispersion for a circularly polarized wave of predefined handedness. Accordingly, retrieved effective medium parameters from simulated results further reveal a difference of 80 in the group indices for left- and right-handed circularly polarized waves at frequencies within the EIT window, which can potentially result in handedness-sensitive pulse delays. These chiral metamaterials which enable a handedness dependent EIT effect may provide more degrees of freedom for designing circular polarization based communication devices.

Electromagnetically induced transparency (EIT), a macroscopic manifestation of quantum coherent interference, has been proposed and demonstrated in multi-level atomic systems. Its characteristic optical signature is a narrow band transparency window in the spectrum of an otherwise absorbing opaque medium^1^. In general, the coherent superposition of states as the precondition of the EIT effect arises from the interaction between light and atomic states, which in general requires some severe conditions such as low temperature, laser pumping, and/or the application of magnetic fields, *etc.* Nevertheless, EIT has been extensively investigated for the past two decades in a variety of different systems^2^,^3^,^4^,^5^,^6^,^7^,^8^,^9^. This keen interest in EIT is mainly because of the extremely steep dispersion properties that are exhibited during matter-light interactions, which may lead to corresponding physical processes such as slow light and enhanced nonlinearities^10^,^11^,^12^,^13^. Meanwhile, the recent development of metamaterials offers unprecedented flexibility in creating electromagnetic resonances for manipulating electromagnetic waves by engineering the properties of their artificial subwavelength “atomic” structure^14^,^15^. Besides exhibiting unconventional material properties, such as negative permeability^16^,^17^, negative refractive index^18^,^19^,^20^, and near-zero-index^21^,^22^, metamaterials have also recently been utilized to demonstrate a classical analogue of EIT through the careful design and arrangement of subwavelength resonating elements^23^,^24^,^25^,^26^,^27^,^28^,^29^,^30^,^31^,^32^,^33^,^34^,^35^,^36^,^37^,^38^. Metamaterial-induced transparency (MIT), taking advantage of the coherent interference between electromagnetic fields induced by radiative (‘bright’) and non-radiative (‘dark’) resonators, enables EIT-like effects at room temperature without the need for other external physical stimuli.

Among the wave properties that can be used to quantify matter-light interactions, polarization is quite unique due to its ability to sense the microscopic and/or macroscopic structure of a medium. Circularly polarized light (CPL) is widely used in optical techniques for the characterization of chiral media, which are ubiquitous in the organic world, *e.g.* sugar enantiomers exhibiting opposite optical activity. However, from the perspective of light manipulation, circular polarization based devices are typically comprised of bulky components, which are necessary in order to detect the weak chiral responses in natural materials. In other words, the handedness-enabled potential of such devices is severely hampered. Recently, chiral metamaterials, artificial arrays of subwavelength resonators that lack mirror symmetry, have been reported to exhibit optical chirality orders of magnitude stronger than that of natural materials. The remarkable chiral response made possible by metamaterials, with a total thickness far less than the operating wavelength, facilitates the ability to strongly manipulate circularly polarized waves^39^,^40^,^41^,^42^,^43^,^44^,^45^,^46^,^47^,^48^,^49^, including negative refraction resulting from chirality^50^. Beyond the linear regime, chiral metamaterials have recently been shown to effectively enable enhanced nonlinearities^51^,^52^. Moreover, chiral metamaterials offer the exciting promise of ultracompact devices for polarization control, bio-sensing and spectroscopy, and chiral-sensitive enhanced imaging, to name a few.

Here, we provide the first experimental demonstration of the handedness-sensitive EIT effect in hybrid chiral metamaterials composed of Born−Kuhn type resonators^47^ and split ring resonators (SRRs). In our design, Born−Kuhn type resonators interact with circularly polarized electromagnetic waves to induce a chiral-sensitive non-radiative resonance in nearby split ring resonators (SRRs), which gives rise to the corresponding transparency window of a prescribed handedness. The coherent coupling between the Born−Kuhn type resonators and the SRRs results in an extremely steep dispersion of the circularly polarized waves, which facilitates the realization of a pronounced difference in group velocity for the opposite handedness. Accordingly, this class of hybrid chiral metamaterials can potentially enable ultracompact and handedness dependent delay lines for circularly polarized waves at prescribed frequencies.

## Results

**Figure 1:**
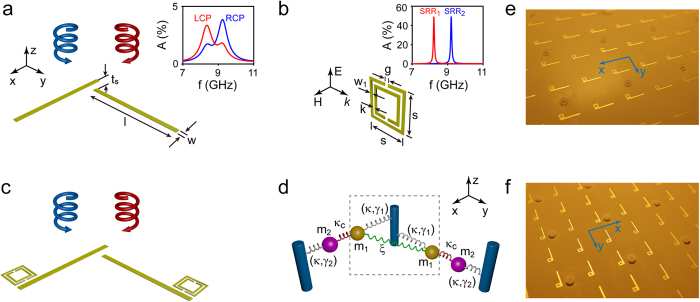
Realization of the electromagnetically induced transparency (EIT) effect in chiral metamaterial systems. (**a**)–(**c**) Schematic of the Born−Kuhn type resonator, magnetic SRR and the subunit of the hybrid chiral metamaterial. Geometrical parameters (all unit in mm): l = 11.5, w = 0.5, t_s_ = 2.28 (substrate: RO3003 (*ε*_r_ = 3, tan*δ* = 0.0013), not shown), the side-length of SRR_1_ and SRR_2_ is 2.68 and 2.47, respectively, and g = w_1_ = k = 0.15. The copper cladding is 35 μm thick. The subunits are arranged in a two-dimensional square lattice with a lattice constant of 20.5 mm. Inset of (**a**) the absorption spectrum for circularly polarized waves at normal incidence on the chiral metamaterial comprising an array of Born−Kuhn type resonators. Inset of (**b**) the absorption spectrum corresponding to a linearly polarized wave normally incident on two different SRR arrays (*i.e.*, SRR_1_ and SRR_2_), both exhibiting a magnetic resonance. (**d**) The mechanical coupled-oscillator model of the hybrid chiral metamaterial system in (**c**), where the damping factors satisfy 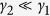. For illustration, a photo of the front and reverse side of the HCMM_1_ sample is shown in (**e**) and (**f**), respectively.

Subunits of systems for MIT are in general composed of two types of resonators which are arranged in an appropriate manner and responsible for the radiative and non-radiative modes respectively. However, for observation of the chiral-sensitive EIT effect, the radiative resonator should primarily be able to support chiral responses. The signature form of the electric field vector for CPL propagating along the z axis, *E*_*x*_ = ±*iE*_*y*_ (where the sign is determined by the handedness), provides a clue for how to achieve strong chiral coupling, *i.e.*, by exploiting two orthogonal harmonic oscillations with a ±*π*/2 phase difference. With this goal in mind, researchers have demonstrated strong chiral responses using a variety of design approaches^39^,^40^,^41^,^42^,^43^,^44^,^45^,^46^,^47^,^48^,^49^. Among them, a structure consisting of twisted cut-wire resonators, which is probably the simplest chiroptical system, has been reported as the realization of the Born−Kuhn model exhibiting optical chirality in the near infrared regime^47^. In addition, supporting both bonding and antibonding resonances under circularly polarized excitations, the Born−Kuhn type resonators are intrinsically radiative due to their coupled-electric-dipole nature. Consequently, in this work, we first design and fabricate an array of Born−Kuhn type resonators, as a chiral metamaterial that exhibits giant chiral responses at X-band microwave frequencies. Figure 1(a) illustrates a schematic of the Born−Kuhn type resonators consisting of two identical cut-wires with a vertical displacement and 90° angular offset. Moreover, the corresponding inset plots the simulated absorption spectra of the metamaterial as a function of frequency, in which an absorption maximum is observed around 8.2 GHz and 9.2 GHz for LCP and RCP waves, respectively. These handedness distinct responses reveal the chirality property of the metamaterial, which will be addressed in detail below. It should be noted that in contrast to that of the plasmonic counterpart, absorption in the printed circuit board (PCB) technology based microwave metamaterials happens within the dielectric substrate (not shown for clarity) rather than in the metallic portions of the structure which can be approximately treated as perfect electrical conductors (PECs).

SRRs have been utilized to realize the sharp non-radiative resonance in various MIT systems^27^,^28^,^29^,^33^,^34^,^35^,^36^ primarily due to the considerable flexibility in engineering their magnetic response. As shown in Fig. 1(b) and its inset, through careful optimization of the geometry of the SRRs (*i.e.*, by varying their outer dimension via the parameter *s* with all other dimensions fixed), a magnetic resonance with a characteristic narrow absorption band can be achieved around either resonant frequency of the chiral metamaterial illustrated in Fig. 1(a). Furthermore, the handedness-dependent EIT effect can be achieved by appropriately arranging the SRR in the near vicinity of either arm of the Born−Kuhn type resonators thereby forming the subunit of the hybrid chiral metamaterial (HCMM), as depicted in Fig. 1(c). We note that the relative position of the SRRs plays a significant role in the resonance coupling. A detailed analysis of this effect has been provided in Ref. 34. Based on this configuration, with no electric resonance expected in the frequency range of concern, the SRRs could not be excited directly by a circularly polarized wave at normal incidence, but instead by the induced field from the Born−Kuhn type resonators. According to EIT theory, the non-radiative resonance should have a much lower damping factor compared to its radiative counterparts, while for MIT systems this condition is equivalent to a quality (Q) factor comparison. Accordingly, defined as Δ*A*/*f*_0_ where Δ*A* represents the full width at half maximum (FWHM), the Q factor associated with the absorption spectrum shown in Fig. 1(a,b) for the Born−Kuhn type resonators under LCP (RCP) excitation is ~8 (12), while that of the SRRs for both geometries is around 180. This indicates that the damping condition required to produce EIT effects is clearly satisfied.

Conventional analog analysis^53^ indicates that the EIT effect is conceivably observable in various classical systems described by a formalism based on similar physics. In addition, the MIT system could be interpreted and visualized through corresponding simple mechanical systems^27^. For a better understanding of the classical analog to our design, in Fig. 1(d) we present the corresponding mechanical model for the subunit of the proposed EIT system comprised of HCMMs. This system can be divided into two components: (i) The pair of gold resonators interrelated by a coupling constant ξ (indicated by the dashed rectangle), which is an analog of the Born−Kuhn type resonators when driven by a harmonic force (not shown) along the x and y axis with a ±*π*/2 phase difference^47^; (ii) The purple non-radiative resonators, represented by the SRRs in our design, which can only be excited by either of the connected gold resonators through *κ*_*c*_. If the damping factors satisfy the condition 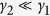, then an EIT-like power spectrum is expected in this mass-spring oscillator system^53^. Hence, this mechanical analogue predicts the chiral-sensitive EIT effect based on the linear coupling between the Born−Kuhn type resonators and the SRRs under LCP and RCP illuminations.

**Figure 2:**
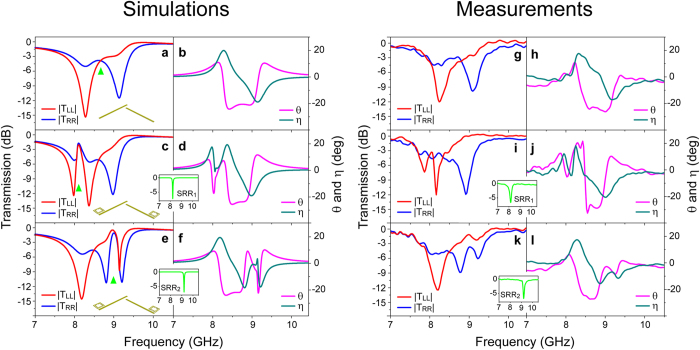
Simulated and measured properties of the proposed chiral metamaterial systems. (**a**), (**c**) and (**e**) Simulated magnitude of the transmission coefficients for the LCP and RCP waves of the Born−Kuhn resonator based chiral metamaterials, hybrid chiral metamaterials with SRR_1_ (s_1_ = 2.68 mm) and SRR_2_ (s_2_ = 2.47 mm). (**b**), (**d**) and (**f**) Corresponding polarization rotation angle (θ) and the resultant ellipticity (η). The insets illustrate the simulated transmission for arrays comprised of SRR_1_ and SRR_2_, respectively. (**g**)–(**l**) Corresponding experimental results obtained from a series of linearly polarized measurements. The insets show responses for the structures comprised of SRR_1_ and SRR_2_ measured by the double monopole antenna method.

To elucidate the handedness dependent EIT characteristics in the proposed metamaterials, we first performed full-wave simulations using the commercial finite integration package CST Microwave Studio. For the chiral metamaterial consisting of an array of Born−Kuhn type resonators (Fig. 1(a)), the transmission spectra of LCP and RCP waves reveal two distinct minima within the prescribed frequency range. This is shown in Fig. 2(a), where 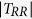 (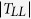) is defined as the amplitude ratio between the RCP (LCP) wave transmitted through the metamaterial and the RCP (LCP) incident wave. A more than 9 dB absolute circular dichroism (CD) (defined as 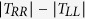) is observed at 8.30 and 9.15 GHz, respectively. Described by the coupled-electric-dipole model, these giant chiral responses^43^,^44^ provide the perfect foundation for further development of the chiral-sensitive EIT effect. As potential candidates for the non-radiative resonators, SRRs of two dimensions (denoted as SRR_1_ and SRR_2_) were optimized to achieve sharp magnetic resonance around the frequencies where the chiral metamaterial experiences resonance under circularly polarized illumination of the opposite handedness, as depicted by the insets of Fig. 2(c–f). In addition, no electric resonance is expected in the frequency domain of concern. Furthermore, Fig. 2(c,e) show the circularly polarized transmission response of the HCMMs with SRRs arranged in the near vicinity of both components of the Born−Kuhn type resonator. For clarity, we refer to the hybrid chiral metamaterials with SRR_1_ and SRR_2_ as HCMM_1_ and HCMM_2_, respectively. Specifically, for the HCMM_1_ (HCMM_2_), a narrow transmission band corresponding to a LCP (RCP) wave with a maximum value of −2.1 dB (−1.1 dB) is observed in the frequency range where the chiral metamaterial originally exhibits a relatively broad stopband for circularly polarized waves of the same handedness. These chiral-sensitive transmission bands (with the frequencies corresponding to maximum transmission indicated by the green triangles in Fig. 2(c,e)), which are closely correlated to the magnetic resonance of the SRRs, reveal the characteristic feature of the EIT effect. On the other hand, for incidence waves with polarization of the opposite handedness, *i.e.*, the RCP wave around 8.10 GHz (LCP wave around 9.00 GHz), a minor transmission peak can also be observed for HCMM_1_ (HCMM_2_). This transmission modulation is indicative of the relevant moderate coupling, which can be attributed to a weak response of the Born−Kuhn type resonator implied by the corresponding high transmission of the chiral metamaterial shown in Fig. 2(a). It also reveals that the introduction of SRRs causes a roughly 150 MHz redshift of the transmission spectra, which possibly arises from the ambient permittivity change due to their electric resonance at higher frequencies (not shown).

Besides the transparency window observed, the coherent interference within the subunit would, on the other hand, simultaneously modulate the microscopic optical responses of the EIT systems. In Fig. 2(b,d,f), we plot the linear polarization rotation angle 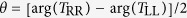 and the ellipticity of the transmitted wave 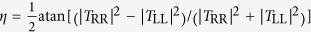 for the proposed chiral metamaterial systems^54^. For the Born−Kuhn type resonator based chiral metamaterial, giant optical activity of −21° (or −317°/λ) is observed around 8.7 GHz, where 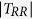 matches 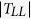 at −3.8 dB as indicated by the green triangle marker in Fig. 2(a). It can be seen that the introduction of SRRs dramatically changes the dispersion of both *θ* and *η* around their well-defined non-radiative resonance. Consequently, we speculate that with the potentially additional narrow bands of pronounced chirality, optical counterparts of the proposed HCMMs could be purposely exploited to offer better performance in sensing and spectroscopy applications.

In the experiment, to obtain the transmission coefficients of the circularly polarized waves (*T*_RR_ and *T*_LL_), four linear transmission coefficients, *i.e.*, *T*_*xx*_, *T*_*yx*_, *T*_*xy*_ and *T*_*yy*_ were measured^44^ for each metamaterial sample of the same configuration consisting of a 14 × 10 array of subunits. It should be noted that in order to match the required substrate thickness, the metallic patterns were printed on only one side of two Rogers RO3003 substrates with a thickness of 1.52 and 0.76 mm, respectively. Nylon screws were placed in the center of every other subunit, where the fields are extremely low, to obtain tightly bounded bi-layer samples. The assembled finite-sized HCMM prototypes, as the sample photos illustrated in Fig. 1(e,f), were then characterized for linearly polarized transmission coefficients (details available in Methods). As shown in Fig. 2(g), two well-separated transmission stopbands are observed for LCP and RCP waves impinging upon the sample composed of Born−Kuhn type resonators, along with a maximum absolute CD of ~7.5 dB at both 8.30 GHz and 9.15 GHz. Further, as illustrated by the insets of Fig. 2(i–l), a narrow band magnetic response characterized by the double monopole antenna method^55^ is identified around 8.20 GHz and 9.20 GHz corresponding to SRR_1_ and SRR_2_, respectively. Moreover, despite the consistent spectral redshift with the simulated results, a high transmission band around 8.00 GHz (9.00 GHz) is observed accordingly for a LCP (RCP) wave transmitted through the chiral EIT system based on HCMM_1_ (HCMM_2_). Compared to the original transmission minima observed in the Born−Kuhn type resonator based chiral metamaterial, a transparency window with 9 dB and 8 dB of transmission enhancement is obtained for LCP and RCP waves, respectively. In addition, *θ* and *η* as a function of frequency extracted from the measured results are plotted in Fig. 2(h,j,l). These results reveal that the experimental data agrees fairly well with the simulations. The relatively moderate transparency windows observed in the experiments are possibly due to two factors arising from uncertainties in the assembly, *i.e.*, a lateral shift between two layers which can cause resonance broadening and an additional interface (air gap) that results in higher losses.

**Figure 3:**
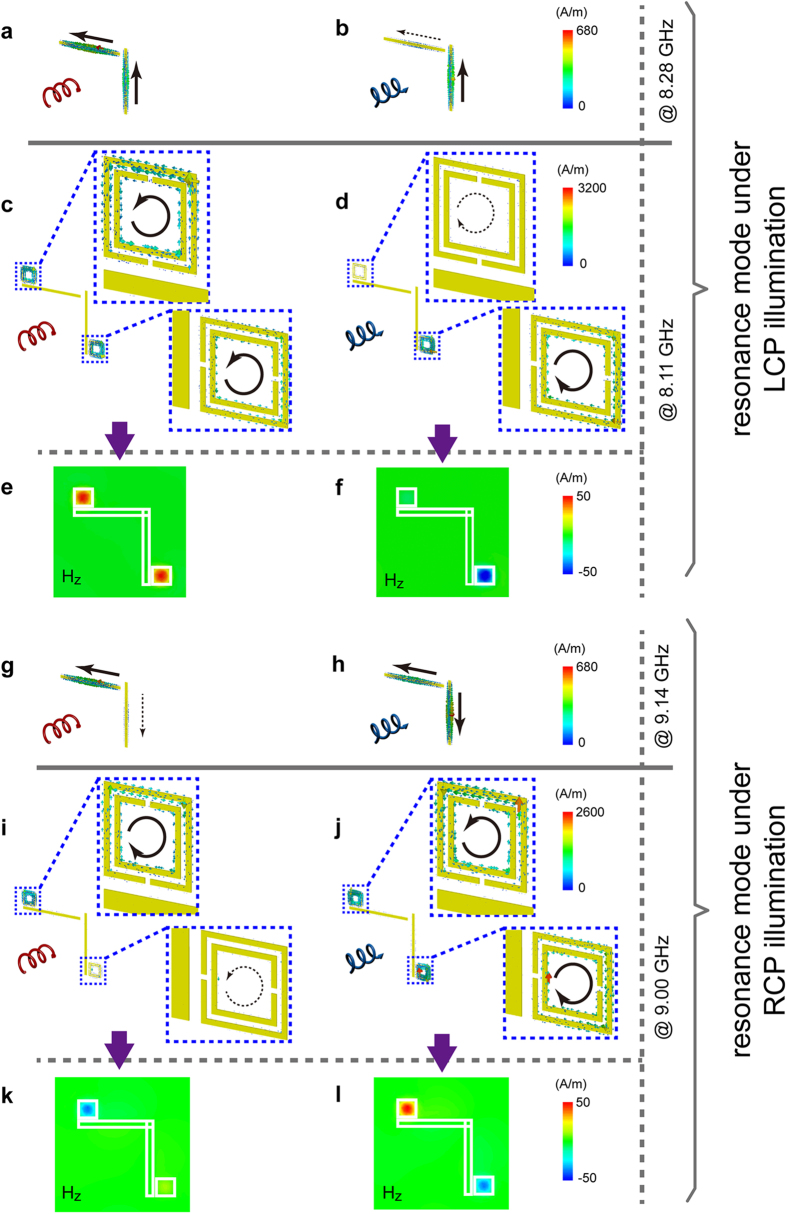
Field analyses of the hybrid chiral metamaterials exhibiting handedness-dependent EIT effects. (**a**) and (**b**) ((**g**) and (**h**)) Induced electric current in the subunit of Born−Kuhn resonator based chiral metamaterial at resonance modes under LCP (RCP) illumination. (**c**) and (**d**) Induced electric current in the subunit of HCMM_1_ at the EIT peak at 8.11 GHz under CPL illumination; (**e**) and (**f**) The corresponding distribution of the z component of magnetic field (H_z_) in a cross-section of the substrate. (**i**) and (**j**) Induced electric current in the subunit of HCMM_2_ at the EIT peak at 9.00 GHz under CPL illumination; (**k**) and (**l**) The corresponding distribution of H_z_ in a cross-section of the substrate. For clarity, the zoomed-in induced current distributions near the SRRs are shown in the insets of (**c**), (**d**), (**i**) and (**j**). The white boxes in (**e**), (**f**), (**k**) and (**l**) indicate the positions of the resonators in their associated subunits.

The chiral-sensitive EIT effect demonstrated here originates from the coherent interference between the radiative and non-radiative resonance within the hybrid chiral metamaterials. This is made evident from the simulated induced current and magnetic field distribution observed in the structures at the critical frequencies, as shown in Fig. 3. First of all, the bonding and antibonding modes are identified from Fig. 3(a,h) at 8.30 GHz and 9.15 GHz, respectively, which is a feature characteristic of a Born−Kuhn type system exhibiting optical chirality^47^. As clearly illustrated in Fig. 3(c), when the HCMM_1_ is illuminated by a LCP wave at 8.11 GHz, strongly induced currents are excited in the SRRs rather than in the Born−Kuhn type resonators, which is further confirmed by the corresponding distribution of the z component (along the direction of incidence) of the magnetic field (H_z_) shown in Fig. 3(e). Similarly, Fig. 3(j,l) illustrate the excitation of SRRs in the HCMM_2_ under illumination of a RCP wave at 9.00 GHz. In brief, the non-radiative resonance mode is excited in both of the SRRs comprising the subunit. On the contrary, as depicted in Fig. 3(d,f,i,k), the induced current and H_z_ confinement can only be effectively excited in one of the subunit’s SRRs, which is responsible for the minor transmission peak around 8.11 GHz (9.00 GHz) produced by a LCP (RCP) wave impinging on the HCMM_1_ (HCMM_2_). Importantly, a closer inspection reveals that the non-radiative resonances represented by the strongly confined H_z_ excited in the two SRRs are in-phase at the EIT peak of LCP illumination (Fig. 3(e)) and out-of-phase for RCP illumination (Fig. 3(l)). Such behavior in the phases of H_z_, which are consistent with the bonding and antibonding resonances indicated in Fig. 3(a,h), unambiguously confirm the coherent nature of the coupling between the Born−Kuhn type resonators and SRRs, as well as the origin of the handedness dependent transparency windows.

**Figure 4:**
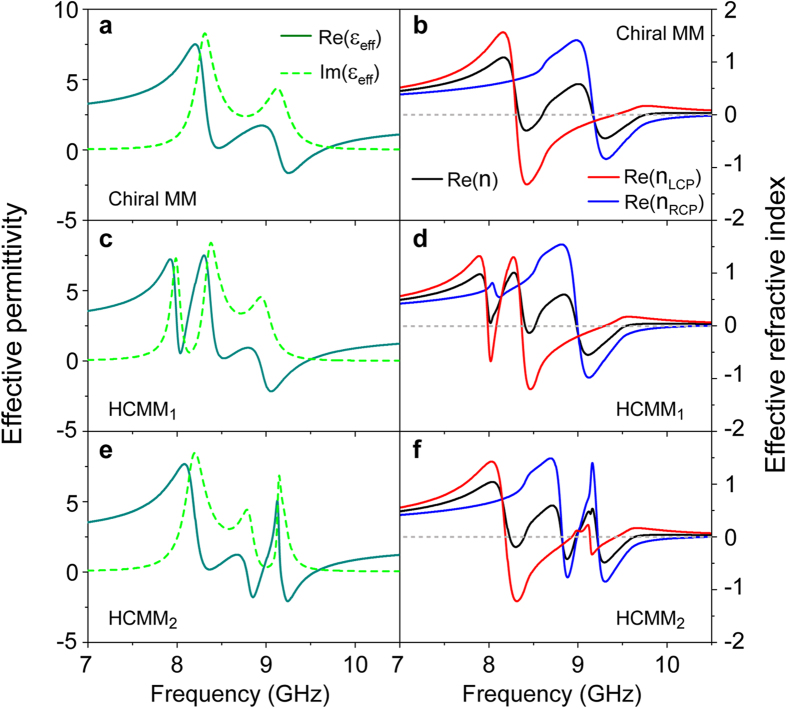
The retrieved effective permittivity and refractive index of the hybrid chiral metamaterials. (**a**), (**c**) and (**e**) the real and imaginary parts of effective permittivity ε_eff_. (**b**), (**d**) and (**f**) the effective refractive index for the LCP and RCP waves and, the refractive index obtained from the relation 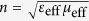, where μ_eff_ is the effective permeability (not shown).

As mentioned earlier, a transparency window appearing in the transmission spectrum of an otherwise opaque medium due to the EIT effect is a manifestation of the coherent process, which possibly results in an extremely steep dispersion relation and other interesting phenomena such as enhanced nonlinearity and the slowing down of light during the process of matter-light interaction. To further understand the EIT effect in chiral systems, we retrieved the effective material parameters of the proposed hybrid chiral metamaterials utilizing the simulated data^56^,^57^. As depicted in Fig. 4(a,c,e), compared to the relatively smooth variation of the effective permittivity of the Born−Kuhn type system, a steeper dispersion slope along with quite low values of Im(ε_eff_) can be seen for either HCMM system corresponding to the frequencies around the transparency windows shown in Fig. 2(c,e). These unique properties suggest that there may be potential applications that would benefit from exploiting the chiral-sensitive EIT effect. Moreover, it has been demonstrated that chiral metamaterials with strong chirality could be an alternative route to achieving a negative refractive index (NRI)^44^,^50^ that can facilitate devices such as a negative phase delay slab, super lensing, and so on. Accordingly, Fig. 4(b) illustrates that the pronounced chirality of the Born−Kuhn type resonator based chiral metamaterial enables NRI for both LCP and RCP waves within the frequency range of interest. More interestingly, as shown in Fig. 4(b,d), a steep dispersion of the refractive index is observed for both of the HCMM systems, which gives rise to an additional band of NRI for circularly polarized waves of relevant handedness. These NRI bands accompanied by the handedness-sensitive EIT effect may be highly sought after due to their intrinsically better figure of merit, *i.e.*, the ratio between the real and imaginary parts of the refractive index (not shown), determined by the highly-desirable ability of the EIT effect to suppress undesirable losses in the system^43^,^44^.

**Figure 5:**
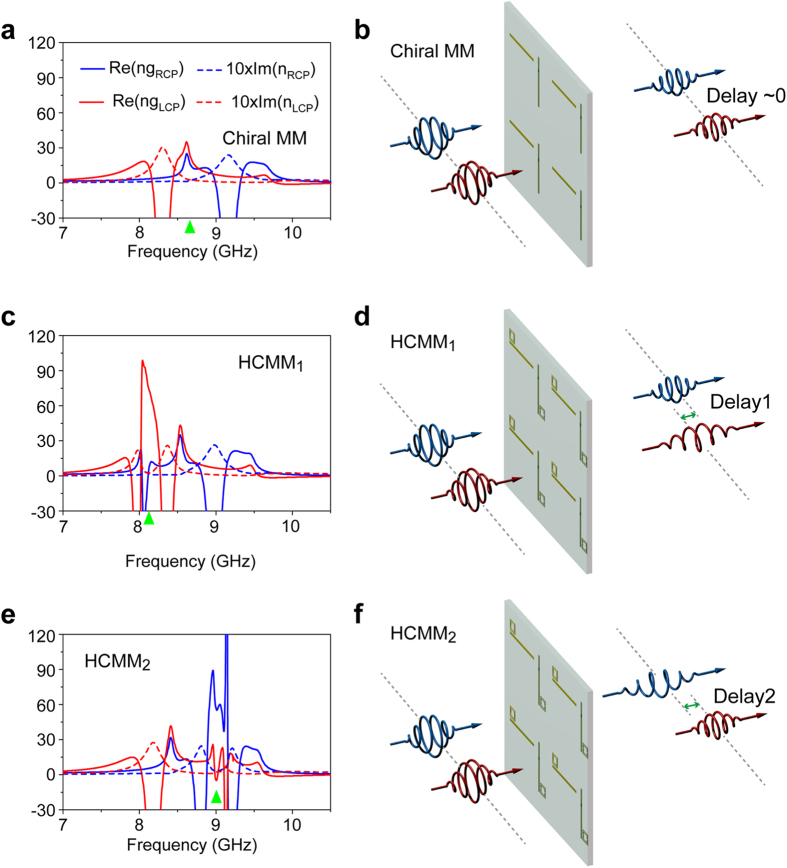
Handedness sensitive slow light in hybrid chiral metamaterials. (**a**), (**c**) and (**e**) Real part of the group index and imaginary part of the refractive index (×10) for LCP and RCP waves in the Born−Kuhn resonator based chiral metamaterials, HCMM_1_ and HCMM_2_. The green triangle in (**a**) indicates the frequency at which 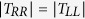 and, those in (**c**) and (**e**) indicate the maximum of the transparency window in Fig. 2(c,e). (**b, d, f**) Schematic illustrating the propagation of pulses consisting of circularly polarized waves of the opposite handedness.

Within the transparency windows associated with the hybrid chiral metamaterials, the dispersion of the refractive index for circularly polarized waves of the opposite handedness behave quite differently. Specifically, a dispersion slope of 9.6/GHz (6.2/GHz) is found in the refractive index for a LCP (RCP) wave around 8.10 GHz (9.00 GHz), while only moderate variation is observed for the other handedness. Consequently, according to the relation *n*_*g*_ = *n* + *ω*d*n*/d*ω* (where *n*_*g*_ and *n* represent the group index and refractive index, respectively), a remarkable difference in group indices (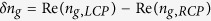, where 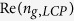 and 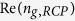 are the real parts of the group index for LCP and RCP waves) is expected for the HCMMs. To elucidate this handedness dependent slow light behavior of the HCMMs, we plot in Fig. 5 the real part of the group index 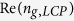 and 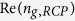 as a function of frequency along with the corresponding imaginary part of the refractive index (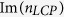 and 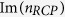, multiplied by a factor of 10). As shown in Fig. 5(a), for the Born−Kuhn type resonator based chiral metamaterial, only a moderate *δn*_*g*_ less than 5 can be found within the low-loss band around 8.70 GHz where the optical activity is the strongest. However, Fig. 5(c) reveals that a sharp peak with a maximum value around 100 is observed in the group index for a LCP wave, *i.e.*, 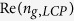. Specifically, a value of 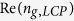 as large as 82 with quite low loss is observed, in contrast to 
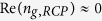
 around 8.10 GHz where the LCP window yields maximum transparency (Fig. 2(c)). Similarly, as shown in Fig. 5(e), within the transparency window for RCP waves (Fig. 2(e)), a dramatic peak in the 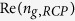 is identified with a value of 60, in contrast to 
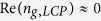
 around 9.00 GHz where 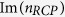 exhibits a low-loss characteristic. The large absolute value of *δn*_*g*_ observed in both HCMM systems may cause distinct delays in the transmitted pulses of the LCP and RCP waves. For instance, suppose we consider a 2.5 ns long Gaussian-shaped LCP pulse centered at 8.10 GHz. Under these conditions the transmitted LCP (RCP) pulse through HCMM_1_ (HCMM_2_), considering a total structure thickness of ~λ/15, will be dramatically delayed by ~27% (~17%) of the pulse width in comparison with a RCP pulse. For the Born−Kuhn type resonator based chiral metamaterial, as a proper reference for a typical delay contrast, roughly 1.6% of the pulse width can be achieved for the circularly polarized 2.5 ns long pulses centered around 8.70 GHz where *T*_RR_ = *T*_LL_ with Δ*n*_*g*_ ≈ 5.

To visualize the handedness dependent slow light effect, we depict in Fig. 5(d,f) the schematics for the transmission of the circularly polarized Gaussian pulses with center frequency at the maximum of the corresponding chiral-sensitive transparency window. The LCP pulse is expected to have a larger delay than the RCP one when transmitting through HCMM_1_ due to the group index contrast, which results in the spatial separation of the transmitted pulses, as illustrated in Fig. 5(d). Similar analysis can be done for the phenomenon shown in Fig. 5(f). Moreover, Fig. 5(b) indicates the near zero spatial delay of pulses with opposite handedness because of the similar values of 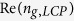 and 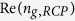 of the chiral metamaterials at the targeted operating frequency. The slow light effect addressed here is closely interrelated with the field trapping phenomena arising from the non-radiative mode excitations illustrated in Fig. 3. We note that these scenarios for controlling pulse delay behavior are provided to help visualize the potential to manipulate circularly polarized waves in the time domain, which is enabled by the proposed hybrid system. The actual pulse propagation can be more complicated due to the steep dispersion around the transparency windows.

## Discussion

Under the framework of metamaterials, the control of resonances and the subsequent electromagnetic wave manipulation are achieved primarily through the geometrical design of subunit resonators. Nevertheless, pronounced tunability has been demonstrated in various types of metamaterial systems based upon quite different mechanisms, as discussed in a recent review paper^58^,^59^. As illustrated in Fig. 2 and the following discussions, the handedness selectivity of the EIT effect realized in the hybrid chiral metamaterial systems is dominated by the non-radiative resonance of the SRRs. Consequently, in the ideal case, it is possible to accomplish the EIT window switching between the opposite handedness by actively controlling the resonant behavior of the SRRs through, for example, loading them with microwave varactors^60^. Furthermore, it is possible to explore the ultrafast dynamics of the group index for circularly polarized waves through the transient loss control mechanism that has been recently demonstrated in metamaterial EIT systems designed to respond to linearly polarized THz radiation^35^. Actually, the EIT phenomenon in metamaterials is also governed by the geometrical coupling conditions^26^. We have observed the continuous variation of the handedness dependent EIT effect due to different locations of the non-radiative resonators. This characteristic might be useful for achieving precise group index control of circularly polarized waves and will be reported elsewhere. In addition, despite the demonstration at microwave frequencies reported in this paper, the proposed chiral-sensitive EIT effect is expected to be present in a broad frequency range including optical. Predictably, at optical wavelengths, the damping factor of the non-radiative resonance that is supported by metallic structures at the nanometer scale, increases dramatically due to the highly dispersive and lossy properties of metals, which would result in broader transparency windows and moderate *δn*_*g*_. However, besides the EIT effect, from the perspective of chiral responses, the chiral-sensitive coherent interference may offer narrow band features to the potential CD spectrum. As a qualitative validation, for HCMM_1_ the Q-factor of the LCP transmission dip around 8.00 GHz (Fig. 2(c)) is ~50, which is roughly 2.5 times higher than that of the transmission dip of the Born−Kuhn type resonator based chiral metamaterial (Fig. 2(a)). In this regard, the handedness dependent coherent coupling between radiative and non-radiative modes in optical HCMM systems might enable a sharp and pronounced spectral response in the CD, which could be beneficial for achieving better sensitivity in chiroptical spectroscopy for structural analyses, as well as for enhanced chiral-sensitive optical nonlinearities.

To summarize, we demonstrate the EIT effect of circularly polarized waves in chiral metamaterial systems, in which the Born−Kuhn type resonators support the chiral responses in a radiative manner and the split-ring resonators (SRRs) support the well-defined non-radiative resonance. Through the introduction of SRRs in the near vicinity of the Born−Kuhn type resonators, we experimentally observed a narrow band transparency window for a circularly polarized wave of prescribed handedness. The non-radiative mode with strong field confinement observed in the hybrid chiral metamaterials (HCMMs) reveals that the origin of the observed EIT phenomena arises from the coherent coupling between the SRRs and the Born−Kuhn type resonators. By extracting the effective material parameters from simulated data, we further illustrate that an extremely steep dispersion relation along with the chiral-sensitive EIT effect enables the giant group index contrast between circularly polarized waves of the opposite handedness. Beyond the circular dichroism enabled functionalities, this EIT effect was shown to facilitate a handedness dependent slow light behavior of the chiral metamaterial, which provides more degrees of freedom in manipulating circularly polarized waves and may have applications in circularly polarized communication systems.

## Methods

### Obtaining the transmission coefficient of circularly polarized waves

According to electromagnetic theory, the eigensolutions for a material with chirality correspond to circularly polarized waves of the opposite handedness, *i.e.*, LCP and RCP waves. In addition, the transmission coefficients of these circularly polarized waves *T*_RR_ and *T*_LL_ can be expressed as a function of four linear transmission coefficients of the chiral medium, *i.e.*, *T*_*xx*_, *T*_*yx*_, *T*_*xy*_ and *T*_*yy*_, such that 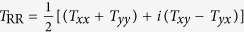 and 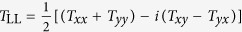. Accordingly, to obtain the transmission coefficients of the circularly polarized waves (*T*_RR_ and *T*_LL_) as shown in Fig. 2, we measured the transmission coefficients (*T*_*xx*_, *T*_*yy*_, *T*_*xy*_ and *T*_*yx*_) associated with linearly polarized waves transmitted through the samples.

### Experimental characterization

Free space transmission measurements of linearly polarized waves were carried out. Two linearly polarized X-band horn antennas were used for transmitting and receiving electromagnetic waves. The two horn antennas were separated by a distance of about 1 meter (roughly 30 times of the operating wavelength) from each other to mimic a plane-wave-like illumination on the samples. The horn antennas were connected to an Agilent E5071C network analyzer for recording the complex transmission coefficients. During the measurement, the transmitting and receiving horns were rotated by 90° accordingly in order to obtain the co-polarized transmission coefficients (*T*_*xx*_ and *T*_*yy*_) and the cross-polarized transmission coefficients (*T*_*yx*_ and *T*_*xy*_), which were normalized to the co-polarized transmission coefficient measured when the sample was absent. The four sets of complex transmission coefficients were then used to further retrieve the transmission coefficients of a circularly polarized waves (*T*_RR_ and *T*_LL_).

## Additional Information

**How to cite this article**: Kang, L. *et al.* Handedness Dependent Electromagnetically Induced Transparency in Hybrid Chiral Metamaterials. *Sci. Rep.* **5**, 12224; doi: 10.1038/srep12224 (2015).
